# Approximate Analytical Approach for Fast Prediction of Microwave Sensor Response: Numerical Analysis and Results

**DOI:** 10.3390/s25185683

**Published:** 2025-09-11

**Authors:** Antonio Cuccaro, Raffaele Solimene, Sandra Costanzo

**Affiliations:** 1 Department of Informatics, Modeling, Electronics and Systems Engineering (DIMES), University of Calabria, 87036 Rende, Italy; sandra.costanzo@unical.it; 2 Department of Engineering, University of Campania Luigi Vanvitelli, 81031 Aversa, Italy; raffaele.solimene@unicampania.it

**Keywords:** microwave sensors, antennas, electromagnetics, propagation, measurements

## Abstract

In medical applications, microwave sensors are usually employed to work in direct contact with the human body, therefore requiring an accurate prediction of the electromagnetic interactions with biological tissues. While full-wave simulations can be useful to achieve the above task, they are computationally expensive, especially for iterative sensor optimization. Analytical models may offer a more efficient alternative, but they are often complex, and they must be formulated in a practical way to be useful. As a result, approximate approaches can be advantageous. Traditional approaches, such as plane-wave approximations and transmission-line models, often fail to capture key sensing features. This paper presents an approximate analytical model for standard-aperture sensor configurations to predict the sensor response in terms of the reflection coefficient when placed above a layered medium. The model is based on the assumption that the electromagnetic interaction is primarily governed by the sensor’s dominant mode. Full-wave simulations in the 2–3 GHz frequency range (relevant for medical applications) demonstrate strong agreement with the analytical model, thereby validating its effectiveness as a first-order approximation for sensor–tissue interactions. This provides a reliable and computationally efficient tool to properly manage microwave sensors design in medical applications.

## 1. Introduction

Microwave sensors (MSs) have emerged as a promising technology for a wide range of medical applications, offering a non-invasive, real-time approach to monitoring and diagnosing various health conditions [1,2,3]. These sensors are utilized in diverse fields, including breast cancer detection [4,5], brain stroke monitoring [6,7], liver tumor thermal ablation [8], and blood glucose level estimation [9,10]. Microwave-based techniques offer distinct advantages, such as continuous, non-ionizing monitoring, making them a viable option for long-term patient monitoring without the associated risks of ionizing radiation.

Despite the growing interest in microwave sensor-based systems, one of the major challenges in this field is understanding the complex interactions between the microwave signal and biological tissues. These interactions are highly dependent on several factors, such as the tissue’s dielectric properties, the sensor’s operating frequency, and the sensor’s configuration. Accurately predicting these interactions is crucial in order to anticipate the sensor’s response and, ultimately, for the development of efficient and reliable medical devices.

Currently, full-wave numerical simulations are often employed to model these sensor–tissue interactions. Although these simulations are accurate, they can be computationally expensive and time-consuming, especially when an iterative procedure is required for sensor optimization or the testing of multiple configurations under varying conditions. This computational burden can limit the efficiency of the sensor analysis and design process.

To address these challenges, the development of analytical models that accurately capture sensor–tissue interactions is of great interest. These models can more efficiently predict the electromagnetic behavior of microwave sensors interacting with biological tissues. Indeed, a well-designed analytical model would not only streamline the sensor design process but also serve as a valuable tool for sensor optimization, enabling the evaluation of different sensor configurations and operating conditions before conducting more resource-intensive simulations or physical experiments.

To realize the advantages of analytical models, they must be simple enough to reduce computational load. This drives the need for approximate models that can serve as “first-order” approximations for sensor responses, providing a more computationally efficient means to analyze sensor–tissue interactions. For example, in [8,11], a plane-wave propagation model is used within a 1D planar medium to determine the optimal features of the coupling medium. Other contributions have used the transmission-line model to provide criteria and guidelines for selecting specific materials or metasurface structures as matching media in biomedical microwave applications [12,13]. However, these models generally do not take into account the sensor structure itself, which means that the design rules derived from the transmission-line model require refinement through full-wave simulations [14]. In fact, as suggested by the results presented in [15,16,17], the coupling medium must be carefully chosen to match both the specific sensor used and the biological tissues. This emphasizes the need to develop analytical models, although approximate, that establish a clearer connection between the sensor and the tissues. In particular, the aim is to provide an analytical model able to represent a guideline (the first step) for the design of microwave sensors in medical applications, thereby avoiding more expensive full-wave analysis, which can be used as refinement step for the sensor design.

Building on these arguments, this paper presents an analytical model for an arbitrary aperture sensor configuration in contact with a multilayered dielectric medium. Specifically, the admittance at the sensor aperture is computed under the assumption that only the dominant mode propagates into the waveguide sensor. Explicit expressions for the admittance are derived and examined in detail for both coaxial and rectangular waveguides. The numerical results, which compare the analytical model with full-wave simulations across different scenarios, demonstrate that, although approximate, the analytical model returns results in good agreement with the full-wave simulations. Hence, it can be used for design purposes. Also, since the analytic model provides an explicit link between the data and the tissues’ properties, it can be potentially exploited for the diagnostic stage by addressing the related inverse problem.

## 2. Waveguide Sensor: Mathematical Model

In this section, we introduce the approximate analytical model for estimating the input admittance of an arbitrary cross section-shaped waveguide sensor.

**Figure 1:**
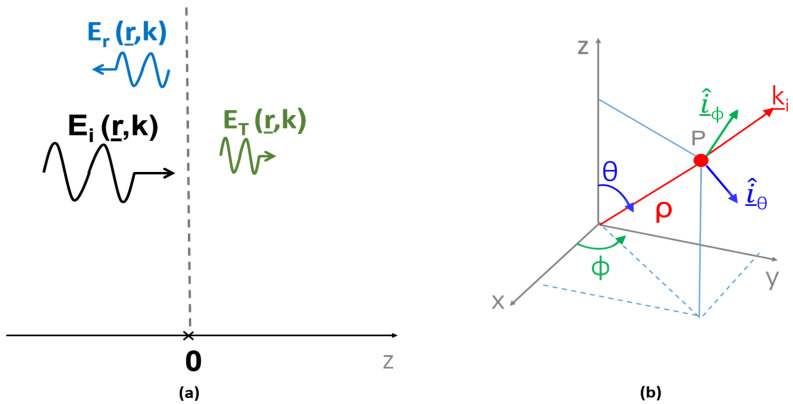
Panel (**a**) highlights the incident, reflected, and transmitted field with reference to the plane (z=0). Panel (**b**) shows the spherical reference frame employed in Equation (3).

To this end, let us begin by considering the simpler half-space geometry depicted in Figure 1a. An incident field propagating along the positive $\hat{z}$ direction impinges on the medium discontinuity located at z=0, giving rise to the reflected and transmitted fields. The incident field, expressed in terms of its plane-wave spectrum (PWS) expansion, can be represented as follows:

(1) $${\underline{E}}_{i}\left(\underline{r},k\right)=\int_{-\infty }^{+\infty }{\underline{\hat{E}}}_{i}\left({\underline{k}}_{t}\right)e^{j\underline{k}\cdot \underline{r}}d{\underline{k}}_{t}$$

and

(2) $${\underline{H}}_{i}\left(\underline{r},k\right)=\int_{-\infty }^{+\infty }{\underline{\hat{H}}}_{i}\left({\underline{k}}_{t}\right)e^{j\underline{k}\cdot \underline{r}}d{\underline{k}}_{t},$$

where $\underline{r}={\underline{r}}_{t}+z\hat{z}$ is the field point, with ${\underline{r}}_{t}=\left(x,y\right)$; $k=\omega \sqrt{\epsilon \mu }$ is the medium wave number in the half-space (z<0), assumed to be homogeneous; and ω is the angular frequency. ${\underline{\hat{E}}}_{i}$ and ${\underline{\hat{H}}}_{i}$ are the PSWs of the electric and magnetic fields, respectively, and ${\underline{k}}_{t}=\left(k_{x},k_{y}\right)$ and $\underline{k}={\underline{k}}_{t}+k_{z}\hat{z}$, with

(3) $$\begin{array}{cc} \; & k_{x}=k\sin\theta \cos\phi \\ \; & k_{y}=k\sin\theta \sin\phi \\ \; & k_{z}=k\cos\theta \end{array}$$

It is convenient to express the PWSs as follows:

(4) $${\underline{\hat{E}}}_{i}\left({\underline{k}}_{t}\right)={\hat{E}}_{TE}\left({\underline{k}}_{t}\right){\hat{i}}_{\phi }+{\hat{E}}_{TM}\left({\underline{k}}_{t}\right){\hat{i}}_{\theta }$$

and

(5) $${\underline{\hat{H}}}_{i}\left({\underline{k}}_{t}\right)=Y\left[{\hat{E}}_{TM}\left({\underline{k}}_{t}\right){\hat{i}}_{\phi }-{\hat{E}}_{TE}\left({\underline{k}}_{t}\right){\hat{i}}_{\theta }\right],$$

where $Y=\sqrt{\frac{\epsilon }{\mu }}$ is the medium admittance. Equations (4) and (5) decompose the field PWS into its transverse electric (TE) and transverse magnetic (TM) components, respectively; Figure 1b helps in clarifying the meaning of unitary vectors appearing in the previous two equations, corresponding to orthogonal and parallel polarizations, respectively.

**Figure 2:**
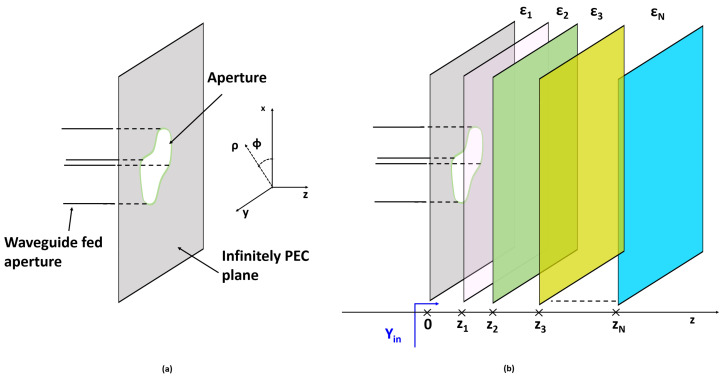
Pictorial view of the considered problem. Panel (**a**) shows the waveguide-fed aperture. In particular, it is assumed that the waveguide aperture occurs in a metallic screen. Panel (**b**) shows the waveguide in contact with a layered medium at z=0. $Y_{in}$ is the wave admittance at the layered medium interface.

In view of the actual problem under consideration (see Figure 2), it is convenient to recast Equation (5) using a cylindrical reference frame, where *z* is the longitudinal axis. Accordingly, the plane-wave spectrum representation of the incident field can be conveniently rewritten as follows:

(6) $${\underline{\hat{E}}}_{i}\left({\underline{k}}_{t}\right)={\hat{E}}_{TE}\left({\underline{k}}_{t}\right){\hat{i}}_{\phi }+{\hat{E}}_{TM}\left({\underline{k}}_{t}\right)\cos\theta {\hat{i}}_{\rho }-{\hat{E}}_{TM}\left({\underline{k}}_{t}\right)\sin\theta {\hat{i}}_{z}$$

and

$${\underline{\hat{H}}}_{i}\left({\underline{k}}_{t}\right)=Y\times$$

(7) $$\left[{\hat{E}}_{TM}\left({\underline{k}}_{t}\right){\hat{i}}_{\phi }-{\hat{E}}_{TE}\left({\underline{k}}_{t}\right)\cos\theta {\hat{i}}_{\rho }+{\hat{E}}_{TE}\left({\underline{k}}_{t}\right)\sin\theta {\hat{i}}_{z}\right].$$

Note that since we are now considering the field propagating inside the guide, *Y* represents the admittance of the medium filling the waveguide.

The reflected and transmitted field PWSs (due to the discontinuity at z=0) can be easily determined and expressed in terms of the Fresnel reflection coefficients as follows:

(8) $$\begin{array}{cc} \; & {\underline{\hat{E}}}_{r}\left({\underline{k}}_{t}\right)=[{\hat{E}}_{TE}\left({\underline{k}}_{t}\right)\Gamma_{TE}\left({\underline{k}}_{t}\right){\hat{i}}_{\phi }+\\ \; & {\hat{E}}_{TM}\left({\underline{k}}_{t}\right)\Gamma_{TM}\left({\underline{k}}_{t}\right)\cos\theta {\hat{i}}_{\rho }+{\hat{E}}_{TM}\left({\underline{k}}_{t}\right)\Gamma_{TM}\left({\underline{k}}_{t}\right)\sin\theta {\hat{i}}_{z}] \end{array}$$

and

(9) $$\begin{array}{cc} \; & {\underline{\hat{H}}}_{r}\left({\underline{k}}_{t}\right)=Y[-{\hat{E}}_{TM}\left({\underline{k}}_{t}\right)\Gamma_{TM}\left({\underline{k}}_{t}\right){\hat{i}}_{\phi }+\\ \; & {\hat{E}}_{TE}\left({\underline{k}}_{t}\right)\Gamma_{TE}\left({\underline{k}}_{t}\right)\cos\theta {\hat{i}}_{\rho }+{\hat{E}}_{TE}\left({\underline{k}}_{t}\right)\Gamma_{TE}\left({\underline{k}}_{t}\right)\sin\theta {\hat{i}}_{z}], \end{array}$$

where $\Gamma_{TE}$ and $\Gamma_{TM}$ denote the reflection coefficients for the TE and TM case, respectively. Hence, the total field at the aperture (i.e., at $z=0^{+}$) can be written as

$$\underline{E}\left(\underline{r},k\right)=\int_{-\infty }^{+\infty }\left[{\underline{\hat{E}}}_{i}\left({\underline{k}}_{t}\right)+{\underline{\hat{E}}}_{r}\left({\underline{k}}_{t}\right)\right]e^{j{\underline{k}}_{t}\cdot {\underline{r}}_{t}}d{\underline{k}}_{t}$$

and

(10) $$\underline{H}\left(\underline{r},k\right)=\int_{-\infty }^{+\infty }\left[{\underline{\hat{H}}}_{i}\left({\underline{k}}_{t}\right)+{\underline{\hat{H}}}_{r}\left({\underline{k}}_{t}\right)\right]e^{j{\underline{k}}_{t}\cdot {\underline{r}}_{t}}d{\underline{k}}_{t}$$

and need to be matched to the field from the waveguide side (i.e., $z=0^{-}$), that is,

$${\underline{E}}_{AP}\left(\underline{r},k\right)=\int_{-\infty }^{+\infty }\left[{\underline{\hat{E}}}_{APi}\left({\underline{k}}_{t}\right)+{\underline{\hat{E}}}_{APr}\left({\underline{k}}_{t}\right)\right]e^{j{\underline{k}}_{t}\cdot {\underline{r}}_{t}}d{\underline{k}}_{t}$$

and

(11) $${\underline{H}}_{AP}\left(\underline{r},k\right)=\int_{-\infty }^{+\infty }\left[{\underline{\hat{H}}}_{APi}\left({\underline{k}}_{t}\right)+{\underline{\hat{H}}}_{APr}\left({\underline{k}}_{t}\right)\right]e^{j{\underline{k}}_{t}\cdot {\underline{r}}_{t}}d{\underline{k}}_{t}$$

Equation (11) establishes the sought-after link between the forward and backward propagating fields inside the waveguide and the properties of the medium in the half-space (z>0; note that the medium can be inhomogeneous along *z*). This equation can then be used to derive the input admittance. Here, we aim to develop an approximate model and simplify the problem by considering only the dominant mode inside the waveguide field. Accordingly, the admittance at the waveguide aperture can be written as [18]

(12) $$Y_{AP}=\frac{1}{|V_{0}{|}^{2}}\int_{AP}\left[{\underline{E}}_{AP}^{*}\left(x,y\right)\times {\underline{H}}_{AP}\left(x,y\right)\right]\cdot {\hat{i}}_{z}dxdy,$$

where $V_{0}$ is the complex (incident plus reflected) amplitude of the dominant mode. Employing Parseval’s identity, Equation (12) can be directly expressed in terms of the field PWS as follows:

(13) $$Y_{AP}={\left[\frac{2\pi }{|V_{0}|}\right]}^{2}\int_{-\infty }^{+\infty }\left[{\underline{\hat{E}}}_{APtang}^{*}\left({\underline{k}}_{t}\right)\times {\underline{\hat{H}}}_{APtang}\left({\underline{k}}_{t}\right)\right]\cdot {\hat{i}}_{z}d{\underline{k}}_{t}$$

It must be noted that in Equation (12), only the tangential electric and magnetic PWS components are relevant, which, according to Equations (8) and (9), are explicitly expressed as follows:

(14) $$\begin{array}{cc} {\underline{\hat{E}}}_{APtang}\left({\underline{k}}_{t}\right)= & {\hat{E}}_{APTE}\left({\underline{k}}_{t}\right)\left(1+\Gamma_{TE}\left({\underline{k}}_{t}\right)\right){\hat{i}}_{\phi }+\\ \; & {\hat{E}}_{APTM}\left({\underline{k}}_{t}\right)k_{z}/k\left(1+\Gamma_{TM}\left({\underline{k}}_{t}\right)\right){\hat{i}}_{\rho } \end{array}$$

and

(15) $$\begin{array}{cc} {\hat{\underline{H}}}_{APtang}\left({\underline{k}}_{t}\right)= & Y[{\hat{E}}_{APTM}\left({\underline{k}}_{t}\right)\left(1-\Gamma_{TM}\left({\underline{k}}_{t}\right)\right){\hat{i}}_{\phi }-\\ \; & {\hat{E}}_{APTE}\left({\underline{k}}_{t}\right)k_{z}/k\left(1-\Gamma_{TE}\left({\underline{k}}_{t}\right)\right){\hat{i}}_{\rho }] \end{array}$$

where $k_{z}/k=\cos\theta$.

Inserting Equations (14) and (15) into Equation (13) yields the following:

(16) $$\begin{array}{cc} Y_{AP}= & {\left[\frac{2\pi }{|V_{0}|}\right]}^{2}\int_{-\infty }^{+\infty }{\left|{\hat{E}}_{APtang}\left({\underline{k}}_{t}\right)\cdot {\hat{i}}_{\phi }\right|}^{2}Y_{in}^{TE}\left({\underline{k}}_{t}\right)+\\ \; & |{\hat{E}}_{APtang}\left({\underline{k}}_{t}\right)\cdot {\hat{i}}_{\rho }{|}^{2}Y_{in}^{TM}\left({\underline{k}}_{t}\right)]d{\underline{k}}_{t} \end{array}$$

with

(17) $$Y_{in}^{TE}\left({\underline{k}}_{t}\right)=Yk_{z}/k\frac{1-\Gamma_{TE}\left({\underline{k}}_{t}\right)}{1+\Gamma_{TE}\left({\underline{k}}_{t}\right)}$$

and

(18) $$Y_{in}^{TM}\left({\underline{k}}_{t}\right)=Yk/k_{z}\frac{1-\Gamma_{TM}\left({\underline{k}}_{t}\right)}{1+\Gamma_{TM}\left({\underline{k}}_{t}\right)}$$

When a stratified tissue is considered, Equations (17) and (18) can be easily calculated by employing, for example, the transmission-line formalism [19].

It is worth noting that, unlike the commonly employed purely plane-wave assumption, the formulation in Equation (16) accounts, albeit approximately, for the actual sensor behavior. In particular, the sensor response/performance can be evaluated in terms of the reflection coefficient:

(19) $$\Gamma =\frac{1-y}{1+y}$$

where $y=Y_{AP}/Y_{0g}$ and $Y_{0g}$ is the admittance of the fundamental mode propagating within the considered sensor. Moreover, in Equation (16), both admittances ($Y_{in}^{TE}$ and $Y_{in}^{TM}$) depend on the integration variable ($k_{t}$), which identifies the generic propagation constant ($k_{z}$) and, accordingly, the direction (θ). Therefore, the admittance is evaluated by including all oblique incidences rather than only the normal direction (θ=0).

Herein, the coaxial cable and the rectangular waveguide are considered, for which the model in Equation (16) particularizes as detailed below.

### 2.1. Coaxial Waveguide

Consider a coaxial line with inner and outer radii denoted as *a* and *b*, respectively. According to the assumptions outlined in the previous section, it is assumed that the aperture field is attributed solely to the dominant TEM mode, that is,

(20) $${\underline{E}}_{AP_{coax}}\left(\rho ,\phi \right)=\frac{V_{0}}{\sqrt{2\pi \log(b/a)}}\frac{1}{\rho }{\hat{i}}_{\rho }$$

with a≤ρ≤b, 0≤ϕ<2π and the field being null outside the aperture. The electric-field PWS is obtained by Fourier transforming the aperture field. Expressing the spectral variable (${\underline{k}}_{t}$) in terms of the polar coordinates, that is, $|{\underline{k}}_{t}|\;=\;\zeta$ and $∠{\underline{k}}_{t}=\theta$, yields the following:

(21) $$\begin{array}{cc} {\hat{\underline{E}}}_{AP_{coax}}\left(\zeta ,\theta \right) & ={\left[\frac{1}{2\pi }\right]}^{2}\int_{AP}{\underline{E}}_{AP_{coax}}\left(\rho ,\phi \right)e^{-jk\zeta \rho \cos(\phi -\theta )}\rho d\rho d\phi \\ \; & =\frac{j}{2\pi }\frac{V_{0}}{\sqrt{2\pi \log(b/a)}}\frac{J_{0}\left(Y\zeta \right)-J_{0}\left(X\zeta \right)}{k\zeta }{\hat{i}}_{\rho } \end{array}$$

with $J_{0}$ being the Bessel function of zero order, X=kb, and Y=ka. The admittance is then obtained by applying Equation (16), which leads to the following:

(22) $$Y_{AP_{coax}}=\frac{1}{\log(Y/X)}\int_{0}^{\infty }\frac{{\left[J_{0}\left(Y\zeta \right)-J_{0}\left(X\zeta \right)\right]}^{2}}{\zeta }Y_{in}^{TM}d\zeta$$

It is worth noting that Equation (22) generally requires the use of contour integration techniques due to the presence of poles on the real axis. These poles correspond to surface waves confined at the interface discontinuity [19]. However, in the cases of interest here, lossy materials are considered. As a result, the poles shift off the real axis, making the integrand smooth and thereby simplifying the numerical integration [20], which is performed here using a MATLAB routine (version 2024a).

### 2.2. Rectangular Waveguide

For a rectangular waveguide of size a>b the dominant mode is ${\mathrm{TE}}_{10}$, whose electric field is expressed as follows:

(23) $${\underline{E}}_{AP_{rec}}\left(\underline{x},\underline{y}\right)=V_{0}\sqrt{\frac{2}{ab}}\cos\left(\frac{\pi x}{a}\right){\hat{i}}_{y}$$

with $\left|x\right|\leq \frac{a}{2},\left|y\right|\leq \frac{b}{2}$ and the field being null outside the aperture. The Fourier transform of this aperture field is expressed as follows:

(24) $${\hat{\underline{E}}}_{AP_{rec}}\left({\underline{k}}_{t}\right)=V_{0}\sqrt{\frac{ab}{32\pi^{2}}}\frac{\sin(\frac{k_{y}b}{2})}{\frac{k_{y}b}{2}}\frac{\cos(\frac{k_{x}a}{2})}{{\left(\pi /2\right)}^{2}-{\left(\frac{k_{x}a}{2}\right)}^{2}}{\hat{i}}_{x}$$

Then, inserting Equation (24) into Equation (16), the following admittance is obtained:

(25) $$\begin{array}{cc} Y_{AP_{rec}}= & \frac{ab}{8}\int_{-\infty }^{+\infty }{\left[\frac{\sin(\frac{k_{y}b}{2})}{\frac{k_{y}b}{2}}\frac{\cos(\frac{k_{y}b}{2})}{{\left(\pi /2\right)}^{2}-{\left(\frac{k_{x}a}{2}\right)}^{2}}\right]}^{2}\times \\ \; & \left[Y_{in}^{TE}\left({\underline{k}}_{t}\right)\frac{k_{y}^{2}}{k_{x}^{2}+k_{y}^{2}}+Y_{in}^{TM}\left({\underline{k}}_{t}\right)\frac{k_{x}^{2}}{k_{x}^{2}+k_{y}^{2}}\right]d{\underline{k}}_{t} \end{array}$$

As in the case of the coaxial waveguide, the singularities in Equation (25) are avoided when lossy materials are considered.

## 3. Numerical Results

In this section, the analytical model is validated through a series of numerical examples by comparing its results with full-wave simulations performed using CST software. Subsequently, the model is employed for the design of a coupling layer and, finally, for the selection of the permittivity of the medium filling the rectangular waveguide to enhance microwave power penetration within a layered medium.

### 3.1. Checking the Analytical Model

In order to perform numerical validations, the coaxial cable is assumed to have inner and outer radii equal to 1 mm and 3.3 mm, respectively, and to be filled with Teflon, with a relative dielectric permittivity equal to ϵ=2.03. The rectangular waveguide is assumed to be air-filled with dimensions of a=8 cm and b=a/3. In both cases, the parameters are selected to ensure the propagation of the only dominant mode within the assumed frequency range.

To assess the accuracy of the analytical model, two scenarios are considered. In the first one, the waveguide sensors are in contact with a homogeneous half-space exhibiting the properties of skin tissue. In the second one, a two-layered half-space medium composed of skin tissue and fat tissue is examined. Specifically, the skin thickness is set equal to 1 mm, in accordance with average literature values [21], while the fat layer is assumed to be semi-infinite. All human tissues are modeled using the four-pole Cole–Cole model based on the results reported in [22].

**Figure 3:**
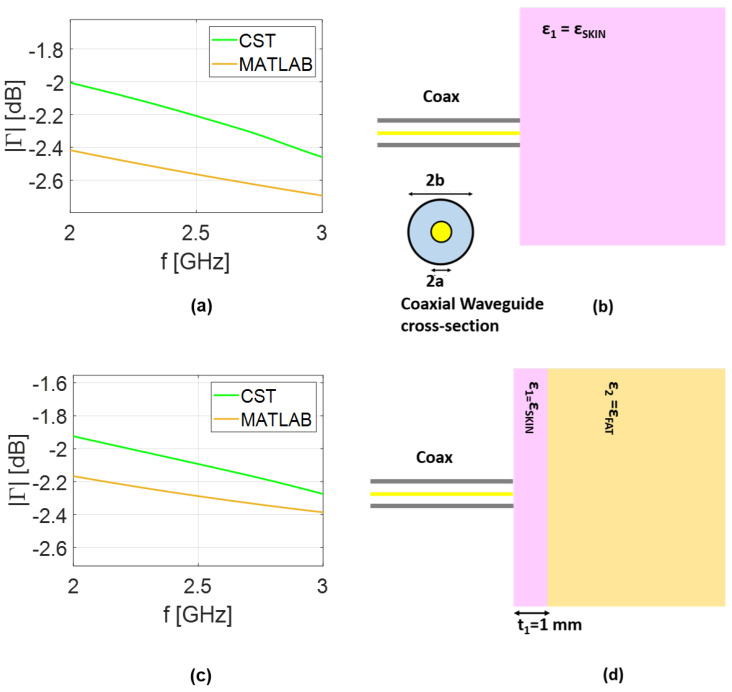
Numerical results: (**a**) reflection coefficient (|Γ|); (**b**) coaxial waveguide of dimensions a=1 mm and b=3.3 mm near half-space phantom; (**c**) reflection coefficient (|Γ|); (**d**) coaxial waveguide near two-layered phantom.

**Figure 4:**
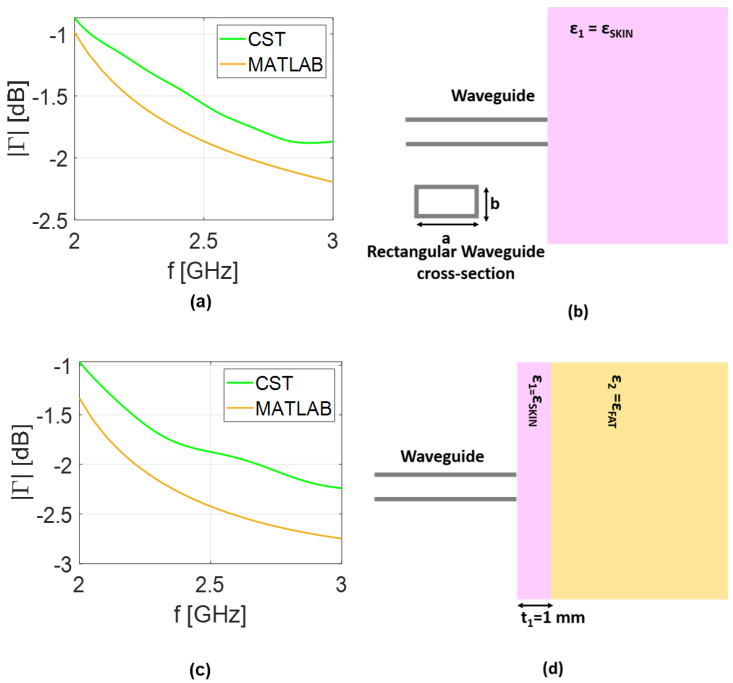
Numerical results: (**a**) reflection coefficient (|Γ|); (**b**) rectangular waveguide with dimensions of a=8 cm and b=a/3 cm near half-space phantom; (**c**) reflection coefficient (|Γ|); (**d**) waveguide near the phantom.

Comparisons between the reflection coefficient predicted by the analytical model and the results obtained in CST simulations is presented in Figure 3 for the coaxial cable and in Figure 4 for the rectangular waveguide. The same scenario adopted in the analytical model is replicated in the full-wave analysis. Therefore, in addition to designing both coaxial and rectangular waveguides, the transverse dimensions of the layered medium are selected to be equal to $2\lambda_{MAX}$, corresponding to the lower frequency band in vacuum, while a thickness of $2\lambda_{MAX}$ was considered to simulate the half-space scenario. Finally, a hexahedral mesh was adopted with a frequency-domain solver with open boundary conditions. As expected, a small mismatch is observed between the analytical model and the CST results, which can be attributed to the approximations made in deriving Equations (22) and (25). Nonetheless, the curves remain in close agreement, differing by only a fraction of a decibel. Including higher-order modes, the number of which, in general, depends on the assumed geometry, can improve the agreement. However, this would complicate the admittance model ($Y_{AP_{rec}}$), reducing its practical usefulness for our purposes. The further advantage of using the analytical model is the speed of analysis. Indeed, for the simplified scenario depicted in Figure 3b, the results took about 30 s using our numerical code. Conversely, the full-wave analysis in CST lasts about 380 s. The gap in simulation times between the models increases for the rectangular waveguide, growing to 70 and 960 s for numerical and full-wave analysis, respectively. This confirms the advantage of the computationally lightweight analytical model as compared to full-wave analysis. All the simulations were performed on an AMD Ryzen 7 laptop with 16 GB RAM.

Previous examples confirm that the analytical model provides sufficiently accurate results. Therefore, it is subsequently used to study the role of a coupling medium which inserted between the sensors and the tissues. Additionally, for the rectangular waveguide case, the model is employed to identify the best filling material to maximize the field intensity inside the biological tissues. Note that, for this second task, we limit our analysis to the rectangular waveguide case, as recent results based on a transmission-line (TL) model are available and can serve as a reference baseline.

### 3.2. Coupling Media Analysis

**Figure 5:**
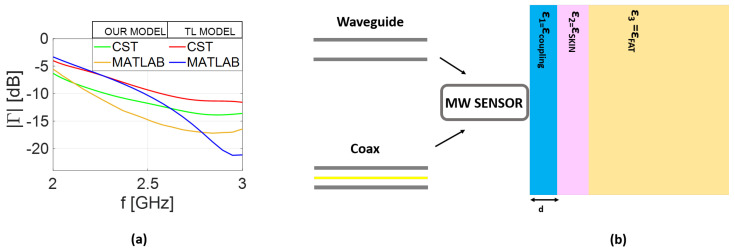
Numerical results: (**a**) reflection coefficient (Γ) and (**b**) coaxial and waveguide sensors near the simplified phantom composed of a coupling layer (thickness of *d*), skin layer (thickness of $t_{1}=1$ mm), and half-space fat. Losses for the coupling medium are fixed at 0.1 [S/m].

Usually, the effect of the coupling medium on the sensor response is applied out by exploiting full-wave analysis or a simplified transmission-line model analysis [13]. However, here, we employ the proposed analytical mathematics as a tool to easily browse across the matching-layer parameters in order to select the most suitable one on a case-by-case basis. The performed numerical analysis refers to the typical scenario considered in medical applications, where a matching layer is inserted between the sensor and the medium under investigation. The geometrical details are reported in Figure 5b, while the coupling media loss is fixed at σ=0.1 [S/m] and the permittivity changes in the range of [1,40]. These values take into account not only the variety of medical applications [15,17,23] but also the practical aspects for the manufacturing of the coupling materia [24].

**Figure 6:**
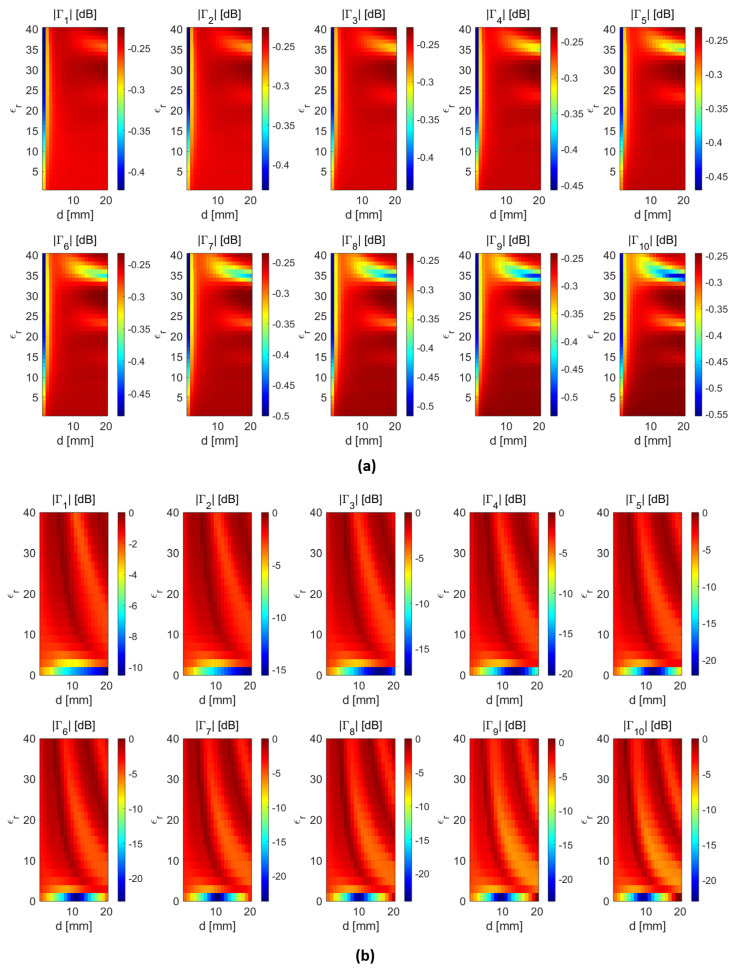
Role of coupling media and numerical reflection coefficient (Γ) estimated by adopting the (**a**) analytical model (Equation (22)) and (**b**) analytical model (Equation (25)). Ten frequencies are uniformly taken in the [2,3] GHz frequency band. The scattering scenario is the same as that depicted in Figure 5b.

The numerical results confirm that, in general, no coupling material can significantly improve the performance of the coaxial waveguide when used as a sensor (see Figure 6a). In contrast, the same figure (panel (b)) suggests that, for the rectangular waveguide case, the sensor response is more sensitive to thickness changes than the permittivity ($\epsilon_{r}$) values (the optimal permittivity value is $\epsilon_{r}=1$) of the coupling media. This is reasonable, since initially, the parameters of the air-filled rectangular waveguide are chosen in order to work in the frequency range of [2,3] GHz. When the structure is used as a sensor, the open end is in direct contact with the biological tissues; then, its performance degrades as shown in Figure 4. However, by properly selecting the thickness (*d*), satisfactory performance can be restored. A reasonable choice is to consider d=10 mm as the optimal thickness, which represents a trade-off between the performance achievable in terms of reflection the coefficient (Γ) and the working frequencies. The previous analysis is confirmed in Figure 5b, where the reflection coefficient (Γ) for the waveguide sensor provided in analytical model (Equation (25)) and full-wave simulations are compared.

**Figure 7:**
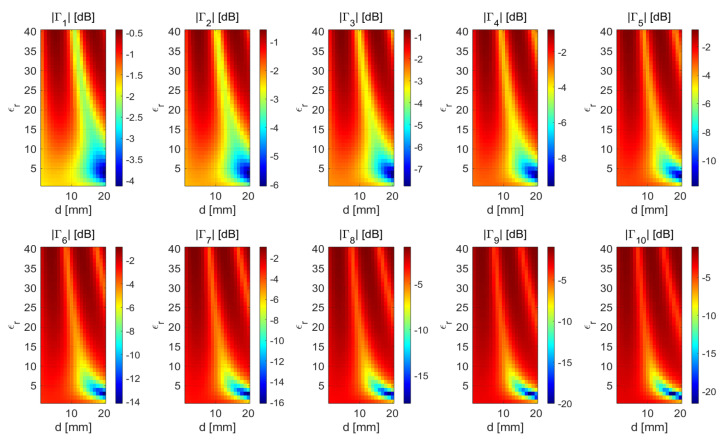
Reflection coefficient (Γ) when the transmission-line model is employed with a rectangular waveguide. The configuration parameters are the same as in Figure 6b.

In Figure 7, the same scenario depicted in Figure 5b is studied by exploiting the (TL) model based on 1D single-plane wave analysis. According to the results, the coupling medium able to improve the rectangular waveguide performance has a permittivity equal to $\epsilon_{r}=3$ and a thickness of d=18 mm. However, by adopting this coupling material, the effective working frequency reduces at a value equal to [2.6,3] GHz (see red solid line in Figure 5a. Moreover, a significant mismatch appears between the results achieved via full-wave analysis and numerical code. Conversely, the analytical model (Equation (25)) provides results similar to those achieved via full-wave analysis. This confirms that the proposed analytical model outperforms the TL method for the prediction of the waveguide sensor behavior.

**Figure 8:**
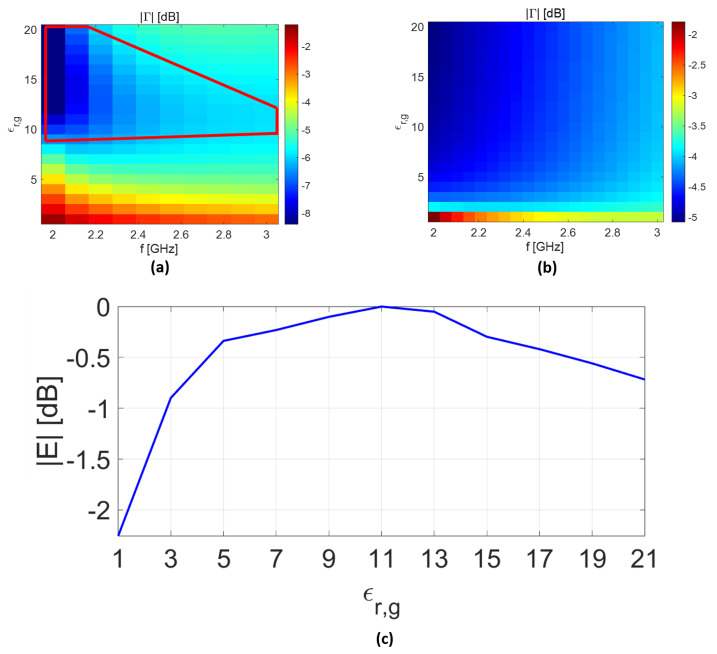
Numerical results: (**a**) reflection coefficient (|Γ|) and (**b**) waveguide near a simplified phantom composed of a coupling layer (thickness of $t_{1}=1$ mm), skin layer (thickness of $t_{2}=1$ mm), and half-space fat. The coupling-medium losses are fixed at 0.1 [S/m]. Panel (**c**) shows the normalized E-field intensity at 10 mm beyond the skin versus the dielectric filled waveguide permittivity variation at 2.5 GHz.

In Figure 8a, the analytical model is employed to identify the best filling material ($\epsilon_{Og}$) so as to maximize the field intensity inside the biological tissues. The scenario of interest is the same as in Figure 4d, except that the rectangular waveguide is filled with material with permittivity changes in the range of [1,21]. Note that for each permittivity value ($\epsilon_{Og}$), the waveguide dimensions are changed accordingly to maintain the same cut-off frequency. As can be seen, a region of acceptable best filling materials is defined (delimited by the red solid line in Figure 8a). Conversely, when the TL model is exploited as in [25], the results seem to confirm that materials with high permittivity provide better performance (see Figure 8b). The validity of the analytical and TL results are checked by implementing the same scattering scenario in CST and measuring the normalized electric field at 10 mm behind the skin layer at 2.5 GHz. As can be seen in Figure 8c, the electric-field intensity reaches the maximum value when $\epsilon_{Og}$ falls in the range of [11,13], confirming the results achieved when the analytical model is exploited. On the contrary, the results predicted by the TL model are less accurate. Indeed, the field intensity inside the biological tissues exhibits opposite behavior compared the results exprected based on Figure 8b.

## 4. Conclusions

A fast analysis method has been presented in this work for waveguide-fed aperture antennas of arbitrary cross-sections, which are adopted to radiate into stratified media for sensing applications. The usefulness of the proposed technique has been illustrated and validated by considering typical examples of practical interest. In particular, the reflection coefficient of a rectangular aperture antenna fed by a rectangular waveguide and radiating into biological tissues has been computed from input admittance knowledge by assuming interesting profiles typical in medical applications.

The validity of the procedure has been checked by adopting a coaxial cable as a sensor.

The achieved numerical results have confirmed that the proposed analytical model allows for prediction of sensor performance in terms of the reflection coefficient, even if in an approximate way. This suggest that the proposed analytical method may be adopted as a useful starting tool to design microwave sensors in medical applications, such as to select the optimum choice of coupling medium. Moreover, the proposed approach is valid in general, and it can be applied for a variety of aperture antenna configurations. Experimental validations are in progress, and they will be the subject of a future extended paper.

## Data Availability

Data are contained within the article.
